# The unblinding of statisticians in clinical trials: commentary on Iflaifel et al., *Trials* 2023

**DOI:** 10.1186/s13063-023-07623-3

**Published:** 2023-09-11

**Authors:** Richard A. Parker

**Affiliations:** Edinburgh Clinical Trials Unit, Usher Institute, University of Edinburgh, Edinburgh, UK

**Keywords:** Clinical trials, RCT, Blinding, Statisticians, Clinical trials unit

## Abstract

Recently, the Blinding of Trial Statisticians research team, Iflaifel and colleagues, have produced detailed guidance regarding the blinding or unblinding of statisticians in clinical trials, based on substantial mixed-methods work. I wish to comment on the research findings. In particular, I argue that open-label trials, non-drug trials, or non-inferiority trials should not be treated any differently from blinded superiority trials with regards to the risk of bias assessment. Prevention of bias should be the priority for definitive randomised controlled trials, regardless of the precise study design.

## Introduction

I read with interest the recent articles published by the Blinding of Trial Statisticians (BOTS) research team regarding blinding statisticians in clinical trials [1, 2]. The BOTS research team have performed an impressive amount of work in relation to the issue of blinding or unblinding statisticians in clinical trials [1]. This is a complex area, with many differing opinions being held and variation amongst trials units. I wish to comment on the key findings from the mixed-methods work published in these two articles: Iflaifel et al. (2022) and Iflaifel et al. (2023) [1, 2].

## The quantitative analysis

Iflaifel et al. (2023) conclude that “No evidence was found to support the assertion that the blinding status of the statistician influenced reported findings” [1]. However, “absence of evidence” does not equate to “evidence of absence”. Based on the logistic regression analysis (152 trial publications), the 95% confidence interval of the odds ratio of a statistically significant trial result ranged from 0.49 to 2.13 [1]. Note that the confidence interval contains odds ratios above 2.0 in favour of a significant result for trials with an unblinded trial statistician relative to those using statistician blinding [1]. Therefore, we can observe from the 95% confidence interval that a wide range of true odds ratios are plausible, and we cannot confidently make a claim in favour of either bias or lack of bias. Furthermore, the authors have not conducted a non-inferiority study; nor have they conducted a randomised experiment. Indeed, there may have been potential residual confounders (e.g. trial size or trial phase) that could have biased the results. Crucially, if there was substantial bias due to unblinding the statistician present in a few studies in the meta-analysis, this is unlikely to have been detected by the conventional statistical analysis approaches used if, for example, the vast majority of studies had no such bias.

## The potential for bias

The BOTS Risk Assessment Tool (BRAT) proposed by the authors is designed to help researchers evaluate the risk of bias and make a decision regarding blinding the statistician in their study [1]. However, even if it is decided that risk of bias is low due to unblinding the trial statistician; bias can still be introduced if the unblinded statistician continues to remain actively involved in decision-making. The problem is that in my experience, a trial statistician will often occupy a powerful position at trial management meetings and rightly will be the “go-to” person to consult by investigators if there are any major changes to study design (e.g. changes to sample size target or changes to outcome measures). If the trial statistician is simultaneously fully unblinded to treatment arm and actively observing the accumulating data from a study, this will at the very least prevent them from taking a fully objective and impartial view at such meetings. I therefore would suggest that there is the *possibility of substantial* bias occurring if a trial statistician is completely unblinded to treatment arm while at the same time is heavily involved in the design, conduct, and running of a clinical trial. An interesting avenue of future research would be to investigate if trials including unblinded trial statisticians are more likely to generate substantial protocol amendments relating to trial design after recruitment begins.

## Type of study design

I agree with Iflaifel et al. (2023) that for certain types of study design, blinding the trial statistician may be of lower priority [1]. For example, feasibility studies usually focus on trial feasibility outcomes such as recruitment, adherence, and retention rates instead of treatment effects, and results are unlikely to be interpreted as definitive. Therefore the impact of any bias in these studies is likely to be less severe. I also agree with the authors that “the resources required to maintain the blind of TSs [Trial Statisticians] need to be proportionate to the perceived benefit, to justify blinding the statistician” [1].

In contrast, blinding of statisticians will be of greatest importance in adaptive trial designs that include formal interim analysis [2]. Maintaining a complete blind of the statistical team is likely to be operationally impossible in such trials. At least one statistician will be needed to conduct the interim analysis, and thus it is likely that they will have clear knowledge of how a treatment is performing during the trial. Hence, without careful use of blinding in the statistical team, the statistician will have the ability (if nothing else) to steer the course of the trial in a certain direction. Clearly, such adaptive trial designs need sufficient resources to be able to properly implement blinding within the statistical team in order to minimise the potential for operational bias [3]. If this is not possible due to scarce resources then arguably adaptive trial designs should not be attempted.

However, that aside, I would question whether some types of design such as open-label trials, non-CTIMP trials or non-inferiority trials need to be treated any differently from blinded CTIMP superiority trials. After all, is not reducing or eliminating bias also important in these trials if their results have the potential to change clinical practice or alter the trajectory of future medical research? This is particularly the case for definitive phase III trials. Whether a trial is open-label or non-CTIMP does not obviate the need to reduce bias in these trials. Iflaifel et al. (2023) suggest that the risk of bias associated with unblinding trial statisticians is likely to be “smaller for open-label trials” on the basis of the focus group findings [1]. The reason presented is that “the nature of the treatments under investigation may not permit blinding and other members of the research team are unblinded” [1]. However, we have to be careful we are not conflating the unblinding of individual patients (which happens in open-label trials or poorly blinded trials) with the unblinding of cumulative summary data or results split by treatment arm. It is necessary and important in open-label trials, as in all trials, that unblinded results split by treatment arm are masked from the investigators during study progress and when writing the statistical analysis plan to avoid bias.

The same can be said for non-CTIMPs. Participants in the qualitative study expressed the view that “it is more important to blind the TS in CTIMPs than in non-CTIMPs” on the basis “of the frequent monitoring and auditing processes conducted by the MHRA” [1, 2]. However, risk of bias would usually be expected to be similar for CTIMP and non-CTIMP trials in general so why treat these trials any differently? The task of blinding statisticians should not be seen as a “vanity project” to be undertaken only if a trial is closely monitored by external observers, but rather as a valid way to reduce the risk of bias being introduced into a study during its progress.

Regarding non-inferiority trials, it has been previously argued in the literature that the extent of the reduction of bias achievable by blinding may be more limited because even a blinded researcher can bias the results to show non-inferiority [4, 5]. On the other hand, Wangge et al. contend that blinding is still important in non-inferiority trials to avoid bias [6]. Indeed, traditionally it has been argued that non-inferiority trials should be conducted with even greater care and rigour than superiority trials [7], and therefore the very idea of being *less* rigorous in the case of statistician blinding seems conflicting. Knowledge of how the trial is performing in terms of establishing non-inferiority (or failing to establish non-inferiority), or knowledge of differences between outcomes, may still introduce serious bias into trial conduct and decision-making. For this reason, I do not agree with the suggestion that “blinding during a superiority trial is more important than in a non-inferiority trial” made in Iflaifel et al. (2022) [2].

## Conclusions

Although I agree that adopting a rigid approach of always blinding the trial statistician might not be appropriate for all trials and in all settings, it is important that we avoid a cavalier approach to unblinding the trial statistician that disregards both the potential risk of bias and the potential severity of any bias. The BRAT tool developed by the authors can help prevent this by providing a framework to aid researchers in assessing the risk of bias. However, I am concerned about the implications of the apparent demotion of certain types of study design (e.g. open-label trials, non-CTIMP, non-inferiority trials) by the authors which may undermine the general need to minimise bias in such designs. Eliminating or minimising bias should always be the priority, and clinical trials units and research teams should be well-supported to achieve this.

## Data Availability

Not applicable.

## Abbreviations

BOTS: Blinding of Trial Statisticians
BRAT: BOTS Risk Assessment Tool
CTIMP: Clinical Trials of Investigational Medicinal Products
MHRA: Medicines and Healthcare products Regulatory Agency
TS: Trial Statistician
